# Clinicopathologic and molecular characterization of stages II-IV gastric cancer with Claudin 18.2 expression

**DOI:** 10.1093/oncolo/oyae238

**Published:** 2024-09-21

**Authors:** Yoonjin Kwak, Tae-Yong Kim, Soo Kyung Nam, Hye Jung Hwang, Daeyoung Han, Hyeon Jeong Oh, Seong-Ho Kong, Do Joong Park, Do-Youn Oh, Hyuk-Joon Lee, Seock-Ah Im, Han-Kwang Yang, Hye Seung Lee

**Affiliations:** Department of Pathology, Seoul National University Hospital, Seoul National University College of Medicine, Seoul, Korea; Department of Internal Medicine, Seoul National University Hospital, Seoul National University College of Medicine, Seoul, Korea; Department of Interdisciplinary Program in Cancer Biology, Seoul National University College of Medicine, Seoul, Korea; Department of Pathology, Seoul National University Hospital, Seoul National University College of Medicine, Seoul, Korea; Medical Affairs, Astellas Pharma Korea Inc., Seoul, Korea; Department of Pathology, Seoul National University Bundang Hospital, Seongnam, Korea; Department of Surgery, Seoul National University Hospital, Seoul National University College of Medicine, Seoul, Korea; Department of Surgery, Seoul National University Hospital, Seoul National University College of Medicine, Seoul, Korea; Cancer Research Institute, Seoul National University College of Medicine, Seoul, Korea; Department of Internal Medicine, Seoul National University Hospital, Seoul National University College of Medicine, Seoul, Korea; Department of Interdisciplinary Program in Cancer Biology, Seoul National University College of Medicine, Seoul, Korea; Cancer Research Institute, Seoul National University College of Medicine, Seoul, Korea; Department of Surgery, Seoul National University Hospital, Seoul National University College of Medicine, Seoul, Korea; Cancer Research Institute, Seoul National University College of Medicine, Seoul, Korea; Department of Internal Medicine, Seoul National University Hospital, Seoul National University College of Medicine, Seoul, Korea; Department of Interdisciplinary Program in Cancer Biology, Seoul National University College of Medicine, Seoul, Korea; Cancer Research Institute, Seoul National University College of Medicine, Seoul, Korea; Department of Surgery, Seoul National University Hospital, Seoul National University College of Medicine, Seoul, Korea; Cancer Research Institute, Seoul National University College of Medicine, Seoul, Korea; Department of Pathology, Seoul National University Hospital, Seoul National University College of Medicine, Seoul, Korea; Department of Interdisciplinary Program in Cancer Biology, Seoul National University College of Medicine, Seoul, Korea; Cancer Research Institute, Seoul National University College of Medicine, Seoul, Korea

**Keywords:** gastric cancer, claudin 18.2, immunohistochemistry, Epstein-Barr virus, microsatellite instability, PD-L1, HER2

## Abstract

**Background:**

Claudin 18.2 (CLDN18.2) is a promising target for targeted therapies in gastric cancer (GC). This study investigated the prevalence of CLDN18.2 expression in patients with stages II-IV GC or gastroesophageal junction (GEJ) adenocarcinoma and its correlation with clinicopathologic features and other crucial GC biomarkers.

**Methods:**

We enrolled 1000 patients diagnosed with stages II-IV GC after surgical treatment. Immunohistochemistry for CLDN18 (43-14A clone), PD-L1 (22C3 pharmDx), HER2, and FGFR2 was performed. CLDN18.2 positivity was defined as moderate-to-strong (2+/3+) membranous staining in ≥75% of tumor cells. CLDN18.2 expression was compared with biomarker expression, Epstein-Barr virus (EBV) association and microsatellite instability status, and clinicopathologic features.

**Result:**

CLDN18.2 was positive in 34.4% of the patients. CLDN18.2 positivity was significantly higher in the middle and upper thirds than in the lower third gastric location (*P* < .001), but there was no correlation with age, sex, or stage (*P* > .05). CLDN18.2 positivity was rare (2.8%) in mucinous adenocarcinoma but frequent (90.9%) in a majority of gastric carcinomas with lymphoid stroma. CLDN18.2 positivity was higher in EBV-associated (*P* < .001) and PD-L1-positive (PD-L1 CPS ≥ 5) GC (*P* = .014) but lower in HER2 positive GC (*P* = .005). CLDN18.2 positivity was not significantly associated with overall survival and disease-free survival.

**Conclusion:**

This study provides a comprehensive evaluation of CLDN18.2 status and its correlation with the clinicopathologic characteristics of patients with stages II-IV GC in Korea and with crucial biomarkers. It may be valuable for guiding future drug development, expanding treatment options, and ultimately improving patient outcomes in GC.

## Teaser

Claudin 18.2 (CLDN18.2) is a promising target for targeted therapies in gastric cancer. This study investigated the prevalence of CLDN18.2 expression in patients with stages II-IV gastric cancer or gastroesophageal junction adenocarcinoma and its correlation with clinicopathologic features and other crucial biomarkers.

**Implications for practice**

Given the potential benefits of CLDN18.2-targeted treatment for gastric cancer (GC), it is important to study CLDN18.2 expression in advanced cases and understand its relation with clinicopathologic features. In Korean stages II-IV GCs, CLDN18.2 expression was observed in 34.4%, suggesting potential-wide applicability of CLDN18.2-targeted therapy. CLDN18.2 was more common in proximal gastric location, PD-L1 positive, and Epstein-Barr virus-positive cases. However, it was rarely seen in mucinous adenocarcinomas. CLDN18.2 expression did not associate with survival outcomes, regardless of stage or chemotherapy use. In patients treated with adjuvant chemotherapy containing fluoropyrimidine ± platinum, there was no survival difference based on CLDN18.2 status.

## Introduction

Claudins (CLDNs) are a family of transmembrane proteins that form the major components of tight junction proteins between cells.^1^ Claudin 18.2 (CLDN18.2) is highly expressed in epithelial tight junctions of normal gastric mucosa.^2^ During malignant transformation, perturbations in gastric mucosa cell polarity and structural loss may result in increased exposure of CLDN18.2, making it more accessible to therapeutics.^3^ Zolbetuximab is an isoform-specific monoclonal antibody that targets the transmembrane protein CLDN18.2, tagging gastric cancer (GC) or gastroesophageal junction (GEJ) cancer cells for destruction by antibody-dependent cellular cytotoxicity (ADCC) and complement-dependent cytotoxicity (CDC).^4^ The global phase III GLOW and SPOTLIGHT trials showed that zolbetuximab combined with chemotherapy as a first-line treatment improved survival in HER2-negative GC or GEJ cancer in patients with CLDN18.2-positive tumors.^5,6^ Given that advanced gastric and GEJ adenocarcinomas are highly aggressive, biomarker profiling is critical for the appropriate diagnosis and therapy. The National Comprehensive Cancer Network recommends trastuzumab combined with chemotherapy for HER2-positive gastric or GEJ adenocarcinoma and nivolumab combined with chemotherapy for HER2-negative and PD-L1 CPS ≥ 5 as first-line treatment.^7^ Therefore, it is important to obtain biomarker data, including prevalence rates and co-expression with other selected GC biomarkers.

In both the SPOTLIGHT and GLOW clinical trials, the prevalence of CLDN18.2-positive cancer was 38.4%.^5,6^ In the SPOTLIGHT trial, 42% of HER2-negative patients showed CLDN18.2-positive status.^6^ However, single-institution studies have reported CLDN18.2 prevalence rates of 14%–88% owing to various testing protocols and interpretations of CLDN18.2 immunohistochemistry (IHC).^8–21^ To date, there are no comprehensive real-world CLDN18.2 biomarker data that could help identify patients who may benefit from treatment with zolbetuximab or other targeted therapies.

GC is a heterogeneous disease that is classified into intestinal, diffuse, mixed, and indeterminate histologic subtypes according to the Lauren classification and into 4 molecular subtypes: Epstein-Barr virus (EBV), microsatellite instability (MSI), chromosomal instability (CIN), and genomically stable (GS) by TCGA (The Cancer Genome Atlas).^22^ Therefore, understanding the prevalence of CLDN18.2 expression in gastric or GEJ adenocarcinoma patients, according to cancer staging, clinicopathologic features, and molecular subtypes, may guide CLDN18.2 testing. A clear correlation between CLDN18.2 expression and clinical or pathologic characteristics and survival data is lacking. Additionally, there are limited prognostic data on CLDN18.2 expression correlating to patient outcomes in the front-line setting when adjuvant chemotherapy is used. Clinicopathologic characterization of CLDN18.2-positivity in gastric or GEJ cancers and its correlation with patient outcomes in a large cohort is warranted to aid patient management.

In this study, we aimed to reveal CLDN18.2 positivity, as defined in clinical trials, in a large cohort of stages II-IV gastric and GEJ adenocarcinomas, to investigate its association with crucial GC biomarkers, and to clarify its correlation with clinicopathologic features and patient survival, particularly through stratification by cancer stage and adjuvant or palliative chemotherapy status.

## Materials and methods

### Patient selection and tissue microarray construction

Patients who underwent surgical resection for stages II–IV gastric or GEJ adenocarcinoma at Seoul National University Hospital (between 2012 and 2014) or Seoul National University Bundang Hospital (between 2006 and 2013) were enrolled to obtain sufficient tissue samples. Patients treated with preoperative chemotherapy or radiotherapy were excluded. Clinicopathologic features, including age, sex, and stage according to the AJCC 8th edition, histologic type according to the WHO 5th edition, histologic grade, tumor location, and histologic type according to Lauren classification, tumor size, lymphatic/venous/perineural invasion, and tumor border, were recorded by reviewing electronic medical charts and pathologic diagnoses. The status of adjuvant or palliative chemotherapy was recorded by reviewing electronic medical charts. Metastatic site information was available in 232 patients diagnosed with distant metastases at initial diagnosis or during postoperative follow-up.

This study was approved by the Institutional Review Board of Seoul National University Hospital (reference: H-2010-179-1171) and Seoul National University Bundang Hospital (reference: B-2401-879-103) and was performed in accordance with the Declaration of Helsinki for biomedical research involving human subjects.

**Figure 1.**
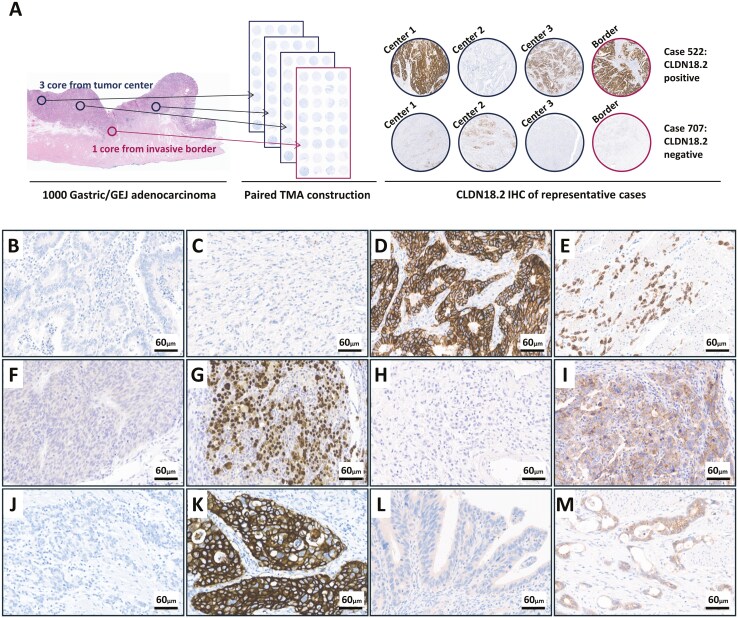
Tissue microarray construction and representative immunohistochemical staining patterns. (A) Schematic illustration of tissue microarray (TMA) construction, demonstrating how the TMA captures intratumoral heterogeneity. (B-M) Representative immunohistochemical staining patterns for various markers, B. CLDN18.2-negative intestinal-type GC. (C) CLDN18.2 negative diffuse-type GC. (D) CLDN18.2-positive intestinal-type GC. (E) CLDN18.2-positive diffuse-type GC. (F) EBV negative. (G) EBV positive. (H) PD-L1 CPS < 5. (I) PD-L1 CPS ≥ 5. (J) HER2 negative. (K) HER2 positive. (L) FGFR2 negative. (M) FGFR2 positive.

Regarding the surgically collected samples, tissue array (TMA) blocks were constructed as previously described (Superbiochips Laboratories, Seoul, Korea). To reflect intratumoral heterogeneity, 4 representative cores (3 from the tumor center and one from the invasive border) with a diameter of 2 mm were obtained from the original formalin-fixed paraffin-embedded (FFPE) block in each case and rearranged into new TMA blocks (Figure 1).

### Immunohistochemistry

Each sample was evaluated by IHC as a negative control without the primary antibody and cytokeratin to validate the quality of the samples. IHC was conducted for CLDN18 (43-14A), HER2 (4B5), PD-L1 (22C3 pharmDx assay), and FGFR2b (SP273). Detailed IHC data are provided in Table 1. CLDN18.2 positivity was defined as ≥75% of tumor cells demonstrating moderate-to-strong membranous staining. Representative immunostaining results are shown in Figure 1.

**Table 1.** The details of immunohistochemistry (IHC).

Biomarker	Primary antibody	Autostainer	Interpretation	Definition of positivity
CLDN18	43-14A (mouse monoclonal, RTU, Ventana Medical system, Tucson, AZ, USA)	Ventana BenchMark ULTRA system	Scoring intensity (negative, weak, moderate, and strong) and area (%)	Moderate-to-strong CLDN18 expression ≥ 75% of tumor cells
HER2	4B5 (rabbit monoclonal, prediluted, Ventana Medical Systems)	Ventana BenchMark ULTRA system	Scoring membranous HER2 staining as 1+, faint staining of ≥10% of the tumor cells; 2+, weak-to-moderate staining in ≥ 10% of the tumor cells; 3+, strong staining in 10% of the tumor cells	IHC 3+ or IHC 2+ and *HER2* amplification by SISH
PD-L1	22C3 pharmDx assay (mouse monoclonal, RTU, Dako, Carpinteria, CA, USA)	DAKO autostainer Link 48	Scoring combined positive score (CPS) with various cutoffs of 1, 5, and 10	CPS ≥ 5
FGFR2b	SP273 (rabbit monoclonal, ×100, abcam, Cambridge, UK)	Ventana BenchMark ULTRA system	Scoring membranous staining as 1+, faint staining of ≥10% of the tumor cells; 2+, weak-to-moderate staining in≥10% of the tumor cells; 3+, strong staining in 10% of the tumor cells	IHC 2+ or IHC 3+

Abbreviations: CLDN18, claudin18; RTU, ready-to-use.

### MSI testing

MSI status was assessed at 5 loci (BAT25, BAT26, D2S123, D5S346, and D17S250) according to the National Institutes of Health guidelines. Briefly, the tumor and paired normal areas were macrodissected from the FFPE blocks, and genomic DNA was extracted. After polymerase chain reaction (PCR) amplification, the PCR products were run on the ABI 3731 automatic DNA sequencer (Applied Biosystems). Tumors with instability at 2 or more, one, or no loci were considered MSI-high (MSI-H), MSI-low (MSI-L), or microsatellite stable (MSS), respectively.

### EBV in situ hybridization

EBV was detected using automated EBV-encoded RNA in situ hybridization (ISH). Briefly, TMA sections were digested and hybridized with a BOND EBER probe (Leica Microsystems, Wetzlar, Germany). Staining of these sections was assessed by evaluating the positivity of the tumor cell nuclei.

### HER2 silver ISH

Automatic dual-color SISH of HER2 was performed using an automatic SISH staining device (BenchMark ULTRA) according to the manufacturer’s instructions for INFORM HER2 DNA and INFORM Chromosome 17 (CEP17) probes (Ventana Medical Systems). Signals from 20 tumor cells were counted and an HER2/CEP17 ratio ≥ 2.0 was classiﬁed as the HER2 gene amplification group.

### Statistical analysis

The correlation of CLDN18.2 with clinicopathologic features was tested using chi-squared or Fisher’s exact test for comparison of categorical variables, Student’s *t* test or ANOVA for parametric test of continuous variables, and the Mann-Whitney or Kruskal-Wallis test for non-parametric test of continuous variables. Overall survival (OS) or disease-free survival (DFS) analyses were performed using Kaplan-Meier survival curves with the log-rank test. Estimates of the medians and 95% CIs were derived for each sample group for the exploratory analysis. All analyses were performed using R v.4.0.3. *P*-value < .05 was considered statistically significant.

## Results

### Patient demographics

Initially, 1049 patients with stages II–IV gastric or GEJ adenocarcinoma were enrolled. Cases that indicated insufficient or inadequate tissue quality from the hematoxylin–eosin (H&E) slide review were excluded from the analysis. The tissue quality was validated by IHC of cytokeratin and negative control slides. Immunostaining was performed on 4 tissue microarray (TMA) cores obtained from different locations of the primary tumor to reflect intratumoral heterogeneity. Positive immunostaining was determined by combining the results from all 4 cores.^17,23,24^ Cases with any missing biomarker results in more than one core, including core loss during the IHC procedure, cores containing less than 100 tumor cells, or severe artifacts, were excluded from further analysis. A total of 1000 patients were included in the final analysis.

Supplementary Table S1 summarizes the clinicopathologic characteristics of the included patients. The cohort comprised 652 males and 348 females, with ages ranging from 21 to 92 years and a median age of 61.0 years. The distribution of the patients’ stages was as follows: 410 patients at stage II (41.0%), 533 patients at stage III (53.3%), and 57 patients at stage IV (5.7%). Radical resection alone or in combination with 5-fluorouracil-based adjuvant chemotherapy was the treatment regimen for all patients with stage II or III disease. All stage IV patients either underwent palliative resection or initially intended to undergo radical resection; however, incidental metastasis was identified. Patients were followed up from the date of surgery to the date of the event (death or recurrence) or the last follow-up for overall survival (OS) and disease-free survival (DFS). The median follow-up was 81.3 months (1.7-137.2) for OS and 79.0 months (0.9-137.2) for DFS.

### Clinicopathologic characteristics and clinical outcomes according to CLDN18.2 status

Among 1000 stages II-IV GC patients, 344 (34.4%) were CLDN18.2 positive. Table 2 shows the correlation between clinicopathologic findings and CLDN18.2 positivity. CLDN18.2 positivity was slightly higher in the earlier stages than in the advanced stages (37.3% in stage II GC, 32.6% in stage III GC, and 29.8% in stage IV), but the difference was not statistically significant (*P* = .246). When categorized according to TNM staging (pT, pN, or pM), there was no significant difference in CLDN18.2 positivity (*P* > .05). According to the World Health Organization histologic classification, mucinous adenocarcinoma had the lowest CLDN18 positivity rate (1/36, 2.8%), whereas gastric carcinoma with lymphoid stroma had the highest positivity rate (10/11, 90.9%; *P* < .001). CLDN18.2 positivity was significantly lower in the lower third but higher in the middle or upper third of the stomach (*P* < .001). CLDN18.2 expression was not significantly correlated with sex, age, tumor size, angiolymphatic invasion, or tumor border (*P* > .05). We also compared distant metastatic sites according to CLDN18.2 status (Supplementary Table S2). Metastatic sites did not differ significantly between CLDN18.2-positive and -negative GCs, including peritoneum (seeding), liver, and distant lymph nodes. However, there were 10 patients with ovarian metastases, and all of them were CLDN18.2-negative cases (*P* = .034).

**Table 2.** Clinicopathologic correlation with CLDN18.2 expression status in GC.

		CLDN18.2		*P* value
		Negative	Positive	
		656 (65.6%)	344 (34.4%)	
Gender				.117
	Male	416 (63.8%)	236 (36.2%)	
	Female	240 (69.0%)	108 (31.0%)	
Age [median (range)]		61.5 (22.0; 92.0)	61.0 (20.0;88.0)	.444
pT				.966
	pT1a	3 (60.0%)	2 (40.0%)	
	pT1b	27 (67.5%)	13 (32.5%)	
	pT2	87 (63.5%)	50 (36.5%)	
	pT3	302 (66.2%)	154 (33.8%)	
	pT4a	217 (65.0%)	117 (35.0%)	
	pT4b	20 (71.4%)	8 (28.6%)	
pN				.171
	pN0	107 (58.5%)	76 (41.5%)	
	pN1	136 (65.7%)	71 (34.3%)	
	pN2	169 (65.5%)	89 (34.5%)	
	pN3a	151 (68.6%)	69 (31.4%)	
	pN3b	93 (70.5%)	39 (29.5%)	
Distant metastasis				.545
	M0	616 (65.3%)	327 (34.7%)	
	M1	40 (70.2%)	17 (29.8%)	
pTNM (1)				.636
	IIA	144 (61.3%)	91 (38.7%)	
	IIB	113 (64.6%)	62 (35.4%)	
	IIIA	164 (67.5%)	79 (32.5%)	
	IIIB	123 (66.5%)	62 (33.5%)	
	IIIC	72 (68.6%)	33 (31.4%)	
	IV	40 (70.2%)	17 (29.8%)	
pTNM (2)				.246
	II	257 (62.7%)	153 (37.3%)	
	III	359 (67.4%)	174 (32.6%)	
	IV	40 (70.2%)	17 (29.8%)	
WHO histologic type				<.001
	Tubular WD	11 (78.6%)	3 (21.4%)	
	Tubular MD	163 (61.0%)	104 (39.0%)	
	Tubular PD	187 (65.6%)	98 (34.4%)	
	Papillary	6 (75.0%)	2 (25.0%)	
	PCC	209 (68.3%)	97 (31.7%)	
	Mixed	44 (60.3%)	29 (39.7%)	
	Mucinous	35 (97.2%)	1 (2.8%)	
	GCLS	1 (9.1%)	10 (90.9%)	
Histologic grade				.128
	WD	13 (81.3%)	3 (18.8%)	
	MD	187 (61.9%)	115 (38.1%)	
	PD	214 (64.5%)	118 (35.5%)	
	PCC	242 (69.1%)	108 (30.9%)	
Location				<.001
	Lower	344 (72.0%)	134 (28.0%)	
	Middle	148 (62.2%)	90 (37.8%)	
	Upper	107 (54.6%)	89 (45.4%)	
	GEJ	21 (75.0%)	7 (25.0%)	
	Entire	36 (60.0%)	24 (40.0%)	
Lauren classification				.262
	Intestinal	233 (62.6%)	139 (37.4%)	
	Diffuse	349 (67.9%)	165 (32.1%)	
	Mixed	74 (64.9%)	40 (35.1%)	
Tumor size [median (range)]		5.0 (1.2;20.1)	5.0 (1.5;19.5)	.198
Lymphatic invasion				1.000
	Absent	203 (65.5%)	107 (34.5%)	
	Present	453 (65.7%)	237 (34.3%)	
Venous invasion				.154
	Absent	507 (64.4%)	280 (35.6%)	
	Present	149 (70.0%)	64 (30.0%)	
Perineural invasion				.065
	Absent	230 (69.7%)	100 (30.3%)	
	Present	426 (63.6%)	244 (36.4%)	
Tumor border				1.000
	Expanding	71 (65.7%)	37 (34.3%)	
	Infiltrative	585 (65.6%)	307 (34.4%)	
Chemotherapy				.650
	Adjuvant	542 (65.1%)	291 (34.9%)	
	Palliative	41 (70.7%)	17 (29.3%)	
	No	73 (67.0%)	36 (33.0%)	

Abbreviations: WD, well differentiated; MD, moderately differentiated; PD, poorly differentiated; PCC, poorly cohesive carcinoma; GCLS, gastric carcinoma with lymphoid stroma; GEJ, gastroesophageal junction.

**Figure 2.**
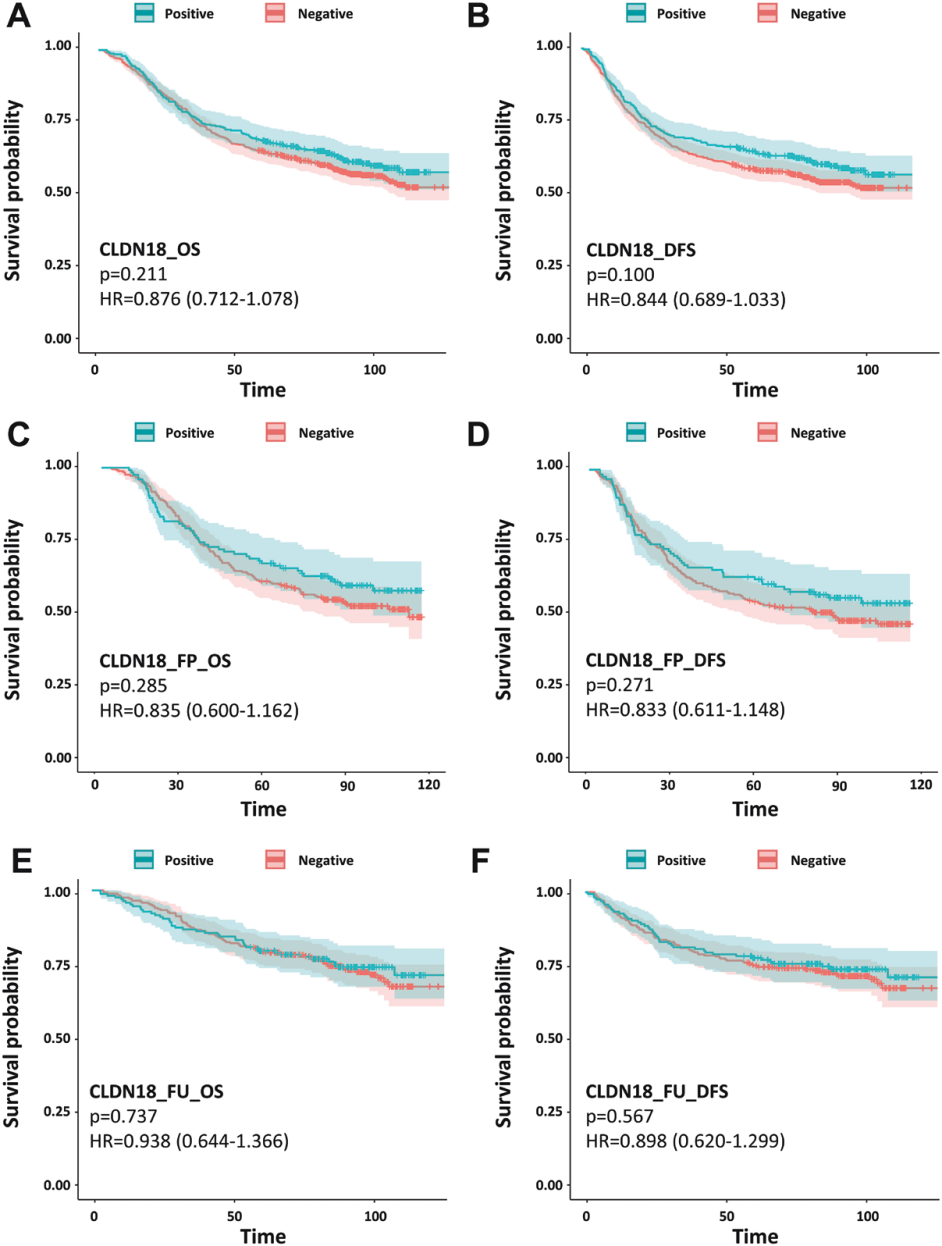
Kaplan-Meier survival curves according to CLDN18.2 expression status. (A) OS in all patients. (B) DFS in all patients. (C) OS in patients treated with fluoropyrimidine and platinum (FP) chemotherapy. (D) DFS in patients treated with FP chemotherapy. (E) OS in patients treated with fluoropyrimidine without platinum (FU) chemotherapy. (F) DFS in patients treated with FU chemotherapy.

Kaplan-Meier survival curves did not reveal any differences in OS and DFS based on CLDN18.2 expression status (Figure 2A and B). When stratified for each AJCC stage, Kaplan-Meier survival curves did not reveal any differences in OS and DFS based on CLDN18.2 expression status (Supplementary Figure S1). In addition, CLDN18.2 expression status was not associated with OS or DFS in patients with GC who received surgery plus adjuvant chemotherapy, surgery plus palliative chemotherapy, or surgery only (Supplementary Figure S2). Furthermore, among the patients with GC treated with adjuvant chemotherapy, there was no survival difference according to CLDN18.2 positivity in the fluoropyrimidine with platinum (Figure 2C and D; *n* = 379) and fluoropyrimidine without platinum groups (Figure 2E and F; *n* = 445).

### Relationship between CLDN18.2 expression and other biomarkers

Among 1000 GC cases, PD-L1 was positive in 367 cases (36.7%), HER2 in 67 cases (6.7%), FGFR2 in 154 cases (15.4%), EBV in 64 cases (6.4%), and MSI in 99 cases (9.9%) (Supplementary Table S3). We analyzed the correlation between the expression of CLDN18.2 and other biomarkers, including PD-L1, HER2, FGFR2, EBV, and MSI (Table 3). CLDN18.2 positivity was slightly higher in PD-L1-positive (CPS ≥ 5:144/367, 39.2%) cases than in PD-L1-negative (CPS < 5:200/633, 31.6%) cases (*P* = .014). Additionally, CLDN18.2 positivity was compared with the various cutoffs for PD-L1 positivity; CLDN18.2 was consistently higher in GC with PD-L1 CPS ≥ 10 (105/260, 40.4%, *P* = .018), PD-L1 CPS ≥ 1 (251/678, 37.0%, *P* = .011) as in CPS ≥ 5 GC. EBV-associated GC and FGFR2-positive GC were consistent with significantly higher CLDN18.2-positive rates (46/64, 71.9% in EBV-associated GC; 68/154, 44.2% in FGFR2 positive GC; *P* < .05). However, CLDN18.2 positivity was less frequently observed in HER2-positive GC (12/67, 17.9%, *P* = .005). CLDN18.2 positivity was similar between microsatellite stable (MSS)/MSI-L GC (34.6%) and MSI-H GC (32.3%) (*P* = .647).

**Table 3.** Correlation between CLDN18.2 expression and other biomarkers.

		Total	CLDN18.2		*P*-value
			Negative	Positive	
		1,000	656 (65.6%)	344 (34.4%)	
PD-L1					.014
	Negative (CPS < 5)	633	433 (68.4%)	200 (31.6%)	
	Positive (CPS ≥ 5)	367	223 (60.8%)	144 (39.2%)	
PD-L1					.018
	CPS < 10	740	501 (67.7%)	239 (32.3%)	
	CPS ≥ 10	260	155 (59.6%)	105 (40.4%)	
PD-L1					.011
	CPS < 1	322	229 (71.1%)	93 (28.9%)	
	CPS ≥ 1	678	427 (63.0%)	251 (37.0%)	
HER2					.005
	Negative	933	601 (64.4%)	332 (35.6%)	
	Positive	67	55 (82.1%)	12 (17.9%)	
FGFR2					.007
	Negative	846	570 (67.4%)	276 (32.6%)	
	Positive	154	86 (55.8%)	68 (44.2%)	
EBV					<.001
	Negative	936	638 (68.2%)	298 (31.8%)	
	Positive	64	18 (28.1%)	46 (71.9%)	
MSI					.647
	MSS/MSI-L	901	589 (65.4%)	312 (34.6%)	
	MSI-H	99	67 (67.7%)	32 (32.3%)	

Abbreviations: MSS, microsatellite stable; MSI-L, microsatellite instability low; MSI-H, microsatellite instability-high.

**Figure 3.**
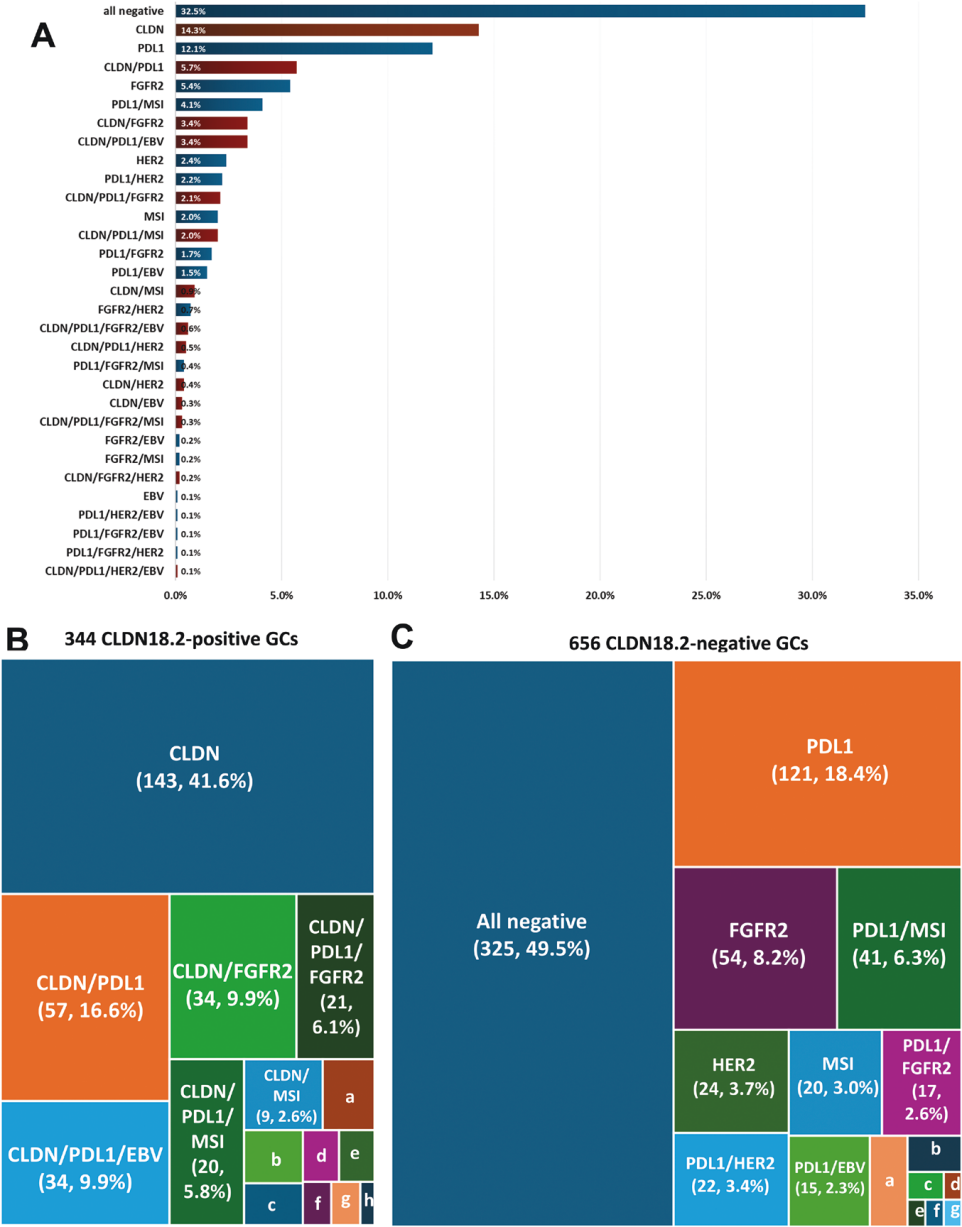
Various combination of CLDN18 and other markers’ co-expression. (A) Frequencies of CLDN18.2, PD-L1, EBV, MSI, HER2, and FGFR2 positivity in a total of 1000 cases of GC. (B) Frequencies of PD-L1, HER2, MSI, EBV, and FGFR2 in 344 CLDN18.2-positive GCs [a, CLDN/PDL1/FGFR2/EBV (6, 1.7%); b, CLDN/PDL1/HER2 (5, 1.5%); c, CLDN/HER2 (4, 1.2%); d, CLDN/PDL1/MSI/FGFR2 (3, 0.9%); e, CLDN/EBV (3, 0.9%); f, CLDN/FGFR2/HER2 (2, 0.6%); g,1 CLDN/FGFR2/EBV (2, 0.6%); h, CLDN/PDL1/EBV/HER2 (1, 0.3%)]. (C) Frequencies of other biomarkers in 656 CLDN18.2-negative GCs [a, FGFR2/HER2 (7, 1.1%); b, PDL1/MSI/FGFR2 (4, 0.6%); c, MSI/FGFR2 (2, 0.3%); d, PDL1/EBV/HER2 (1, 0.2%); e, PDL1/FGFR2/EBV (1, 0.2%); f, PDL1/FGFR2/HER2 (1, 0.2%); g, EBV (1, 0.2%)].

Of the 1000 cases of GC, 325 (32.5%) were negative for all biomarkers (Figure 3A). Of the 1000 cases, 143 cases (14.3%) were positive for CLDN18.2 alone, 121 (12.1%) for PD-L1 (CPS ≥ 5) alone, 54 (5.4%) for FGFR2 alone, 24 (2.4%) for HER2 alone, 20 (2.0%) for MSI-H alone, and 1 patient (0.1%) for EBV alone. Various combination of the other 5 biomarkers (PD-L1, MSI, EBV, HER2, and FGFR2) in CLDN18.2-positive GC and CLDN18.2-negative GC are shown in Figures 3B and C, respectively. In 344 cases positive for CLDN18.2, 143 (41.6%) exhibited exclusive CLDN18.2 expression, while 201 (58.4%) demonstrated co-positivity with other biomarkers. Among CLDN18.2-negative GC cases, approximately half displayed positivity for any biomarker, leaving the other half devoid of biomarker expression. Additionally, rare occurrences were noted in this large cohort, including CLDN18.2-positive/PD-L1-positive/EBV-positive/HER2-positive GC, PD-L1-positive/EBV-positive/FGFR2-positive GC, PD-L1-positive/EBV-positive/HER2-positive GC, and PD-L1-positive/FGFR-positive/HER2-positive GC, with each observed in only one case among the 1000 cases of GC.

## Discussion

CLDN18.2 is a potential candidate for monoclonal antibodies such as zolbetuximab (IMAB362), which are currently undergoing clinical trials for various gastrointestinal malignancies.^16,18,25^ Considering the potential role of this targeted treatment in GC, it is crucial to investigate CLDN18.2 expression in advanced GC cases, understand its relationship with clinicopathologic characteristics, and establish success rates before implementing such targeted therapies. To the best of our knowledge, this is the first study examining CLDN18.2 expression in the largest cohort of 1000 Korean patients with GC. In addition, an essential strength of this study is that we examined CLDN18.2 expression along with other crucial biomarkers through multiple TMA cores to reflect tumor heterogeneity.

In SPOTLIGHT and GLOW, 2 global phase III zolbetuximab trials, the prevalence of CLDN18.2 positivity was approximately 38.4%.^5,6^ In this study, using the same CLDN18 IHC testing method as in the clinical trials and interpretation by an experienced pathologist revealed an overall positive rate of CLDN18 of 34.4%, which is similar to that yielded in the clinical trials. Several single-institution studies have reported CLDN18.2 prevalence ranging from 14% to 88%.^11,12,14,15,17,19^ In the study by Rohde et al, 87% of the patients with GC exhibited CLDN18.2 positivity. This higher positive rate compared to our study may be because their definition of CLDN18.2 positive cases (different intensities and percentage of positive cells) differed from the definition of CLDN18.2 positivity adopted in the phase III zolbetuximab trials.^19^ Furthermore, several studies^9-12,14,15^ have assessed CLDN18.2 expression by employing antibodies different from the CLDN18 clone 43-14A, which was used in phase III trials of zolbetuximab. As we used the same antibody clone and positivity criteria as in previous phase III trials, our findings offer the most reliable real-world data to guide the implementation of zolbetuximab in treating GC.

Recent advances in targeted therapy and immunotherapy have been rapidly applied to the treatment of GC. However, their advantages are primarily confined to the palliative setting,^26^ and there has been no substantial improvement in patient outcomes with new treatments in the adjuvant setting. Furthermore, although the use of neoadjuvant treatments for GC is increasing, they remain restricted to conventional chemotherapy protocols. In this study, we identified a considerable number of CLDN18.2-positive cases with stages II-III GC, indicating the possibility of a broader patient population who could potentially benefit from targeted treatments in both adjuvant and neoadjuvant contexts.

As HER2, PD-L1, and MSI are established biomarkers, and EBV and FGFR2b are emerging biomarkers for biomarker-based therapies in patients with GC,^27^ we compared CLDN18.2 expression status with HER2, PD-L1, MSI, EBV, and FGFR2b. Previous studies have shown varying co-expression rates among relevant biomarkers.^13,17,28^ We found that CLDN18.2 positivity was significantly higher in EBV-associated GC (71.9%), which is consistent with the results of several previous studies.^13,17^ CLDN18.2 positivity was significantly correlated with PD-L1 expression between 37% and 40% regardless of CPS cutoffs (CPS ≥ 1,≥5, and ≥10). Further, no difference in the CLDN18.2 positivity rate was found between MSS/MSI-L and MSI-H GC. In addition, a negative correlation was found between CLDN18.2 expression and HER2 positivity in GC, suggestingthe applicability of CLDN18.2 targeted therapy in HER2-negative GC patients, in accordance with previous studies.^13^ Notably, 143 (41.6%) of the 344 CLDN18.2-positive GC patients did not express any other biomarker. However, several minor groups co-expressed other biomarkers in complex combinations with CLDN18.2, which have not been identified in previous studies. Groups with these minor combinations may have been underrepresented in previous studies that primarily used a single TMA core, which likely did not reflect tumor heterogeneity.

As in previous studies, CLDN18.2 expression was not correlated with the OS of patients with GC in this study. Furthermore, pT, pN, pM, and pTNM stages were not associated with CLDN18.2 expression status. However, we identified several significant correlations between CLDN18.2 expression and the clinicopathologic features. First, a significant relationship was observed between CLDN18.2-positive GC and mid-to-upper third tumor locations in the stomach. Consistent with our findings, Coati et al discovered a significant association between CLDN18.2 expression and non-antral locations.^10^ As GC in Asians is more likely to occur in the distal third, the prevalence of CLDN18.2 in Asians may differ from the global prevalence.^29^ Analysis of the relationship between CLDN18.2 expression and histologic types revealed rare CLDN18.2 positivity (1/36, 2.8%) in mucinous adenocarcinoma, whereas the majority of gastric carcinoma with lymphoid stroma exhibited CLDN18.2 positivity (10/11, 90.9%). Although these are rare histologic types of GC, our results provide a basis for understanding the pathological feature of CLDN18.2-positive GC.

Although this study involved a sizable sample of 1000 Korean patients with GC, there were several limitations. First, it was not a multicenter study, and patients were recruited from only 2 institutions, which is a limitation. The lack of whole-section staining results presents another limitation, but we analyzed the results of 3-4 TMA cores together to reflect as much tumor heterogeneity as possible. Finally, the antibody used for FGFR2 immunostaining was not the same as that used in ongoing clinical trials involving FGFR2.^30^ Additionally, the number of gastroesophageal junction (GEJ) cancer cases in our study was small, representing only 2.8% of the total. This is consistent with the lower prevalence of GEJ cancers in Asian populations compared to Western populations. While this limits the generalizability of our findings to non-Asian populations, our study still provides valuable insights based on a large cohort of Asian patients. Furthermore, we did not evaluate the potential discrepancy in CLDN18.2 expression between primary tumors and metastatic sites. This could be significant as differential expression may influence treatment responses and outcomes. Future studies should aim to address this by including an analysis of CLDN18.2 expression in both primary and metastatic tumor sites to better understand the dynamics of CLDN18.2 in gastric cancer progression and metastasis.

## Conclusion

We evaluated CLDN18.2 expression in Korean patients with stages II-IV GC and found an overall prevalence of 34.4%. CLDN18.2-positive expression was associated with the proximal gastric location, EBV-positive status, and PD-L1 positivity. There were a limited number of patients with overlapping expression of HER2 and CLDN18.2. The results of this study lay the groundwork for exploring the applicability of CLDN18.2-targeted therapy in stages II-III GC patients in the adjuvant/neo-adjuvant setting. The insights from this study may help serve more patients with GC who can benefit from targeted therapies.

## Supplementary material

Supplementary material is available at *The Oncologist* online.

oyae238_suppl_Supplementary_Tables

oyae238_suppl_Supplementary_Material

## Data Availability

The data used and/or analyzed during the current study are available from the corresponding author on reasonable request.
