# Supplementary material for: Clinicopathologic and molecular characterization of stages II-IV gastric cancer with Claudin 18.2 expression
Source: Oncologist. 2024 Sep 21;30(2):oyae238. doi: 10.1093/oncolo/oyae238 (PMC11881060; doi:10.1093/oncolo/oyae238)
Supplement: oyae238_suppl_Supplementary_Tables [file oyae238_suppl_supplementary_tables.docx]

**Supplementary Table S1. Clinicopathologic characteristics of GC patients**

|  | **Variables** | **No. (%)** |
| --- | --- | --- |
| **Total** |  | **1,000 (100%)** |
| gender |  |  |
|  | male | 652 (65.2%) |
|  | female | 348 (34.8%) |
| age |  | 61.0 [20.0;92.0] |
| pT |  |  |
|  | pT1a | 5 (0.5%) |
|  | pT1b | 40 (4.0%) |
|  | pT2 | 137 (13.7%) |
|  | pT3 | 456 (45.6%) |
|  | pT4a | 334 (33.4%) |
|  | pT4b | 28 (2.8%) |
| pN |  |  |
|  | pN0 | 183 (18.3%) |
|  | pN1 | 207 (20.7%) |
|  | pN2 | 258 (25.8%) |
|  | pN3a | 220 (22.0%) |
|  | pN3b | 132 (13.2%) |
| Distant metastasis |  |  |
|  | M0 | 943 (94.3%) |
|  | M1 | 57 (5.7%) |
| pTNM (1) |  |  |
|  | IIA | 235 (23.5%) |
|  | IIB | 175 (17.5%) |
|  | IIIA | 243 (24.3%) |
|  | IIIB | 185 (18.5%) |
|  | IIIC | 105 (10.5%) |
|  | IV | 57 (5.7%) |
| pTNM (2) |  |  |
|  | II | 410 (41.0%) |
|  | III | 533 (53.3%) |
|  | IV | 57 (5.7%) |
| Histologic type by WHO |  |  |
|  | Tubular, WD | 14 (1.4%) |
|  | Tubular, MD | 267 (26.7%) |
|  | Tubular, PD | 285 (28.5%) |
|  | Papillary | 8 (0.8%) |
|  | PCC | 306 (30.6%) |
|  | Mixed | 73 (7.3%) |
|  | Mucinous | 36 (3.6%) |
|  | GCLS | 11 (1.1%) |
| Histologic grade |  |  |
|  | WD | 16 (1.6%) |
|  | MD | 302 (30.2%) |
|  | PD | 332 (33.2%) |
|  | PCC | 350 (35.0%) |
| Location |  |  |
|  | lower | 478 (47.8%) |
|  | middle | 238 (23.8%) |
|  | upper | 196 (19.6%) |
|  | GEJ | 28 (2.8%) |
|  | entire | 60 (6.0%) |
| Histologic type by Lauren |  |  |
|  | intestinal | 372 (37.2%) |
|  | diffuse | 514 (51.4%) |
|  | mixed | 114 (11.4%) |
| size |  | 5.0 [1.2;20.1] |
| Lymphatic invasion |  |  |
|  | absent | 310 (31.0%) |
|  | present | 690 (69.0%) |
| Venous invasion |  |  |
|  | absent | 787 (78.7%) |
|  | present | 213 (21.3%) |
| Perineural invasion |  |  |
|  | absent | 330 (33.0%) |
|  | present | 670 (67.0%) |
| Tumor border |  |  |
|  | expanding | 108 (10.8%) |
|  | infiltrative | 892 (89.2%) |
| Chemotherapy |  |  |
|  | adjuvant | 833 (83.3%) |
|  | palliative | 58 (5.8%) |
|  | no | 109 (10.9%) |

WD, well differentiated; MD, moderately differentiated; PD, poorly differentiated; PCC, poorly cohesive carcinoma; GCLS, gastric carcinoma with lymphoid stroma; GEJ, gastroesophageal junction

**Supplementary Table S2. Comparison of metastatic sites according to CLDN18.2 status.**

|  |  | CLDN18.2 | | total | p value |
| --- | --- | --- | --- | --- | --- |
|  |  | Negative | Positive |  |  |
| peritoneum (seeding) | absent | 64 | 22 | 86 | 0.168 |
|  |  | 40.0% | 30.6% | 37.1% |  |
|  | present | 96 | 50 | 146 |  |
|  |  | 60.0% | 69.4% | 62.9% |  |
| liver | absent | 134 | 64 | 198 | 0.306 |
|  |  | 83.8% | 88.9% | 85.3% |  |
|  | present | 26 | 8 | 34 |  |
|  |  | 16.3% | 11.1% | 14.7% |  |
| distant lymph node | absent | 124 | 56 | 180 | 0.963 |
|  |  | 77.5% | 77.8% | 77.6% |  |
|  | present | 36 | 16 | 52 |  |
|  |  | 22.5% | 22.2% | 22.4% |  |
| ovary | absent | 150 | 72 | 222 | 0.034 |
|  |  | 93.8% | 100.0% | 95.7% |  |
|  | present | 10 | 0 | 10 |  |
|  |  | 6.3% | 0.0% | 4.3% |  |
| bone | absent | 152 | 70 | 222 | 0.728 |
|  |  | 95.0% | 97.2% | 95.7% |  |
|  | present | 8 | 2 | 10 |  |
|  |  | 5.0% | 2.8% | 4.3% |  |
| lung | absent | 156 | 70 | 226 | 1.000 |
|  |  | 97.5% | 97.2% | 97.4% |  |
|  | present | 4 | 2 | 6 |  |
|  |  | 2.5% | 2.8% | 2.6% |  |
| gastric recur | absent | 154 | 68 | 222 | 0.505 |
|  |  | 96.3% | 94.4% | 95.7% |  |
|  | present | 6 | 4 | 10 |  |
|  |  | 3.8% | 5.6% | 4.3% |  |
| leptomeningeal | absent | 158 | 72 | 230 | 1.000 |
|  |  | 98.8% | 100.0% | 99.1% |  |
|  | present | 2 | 0 | 2 |  |
|  |  | 1.3% | 0.0% | 0.9% |  |
| adrenal | absent | 159 | 72 | 231 | 1.000 |
|  |  | 99.4% | 100.0% | 99.6% |  |
|  | present | 1 | 0 | 1 |  |
|  |  | 0.6% | 0.0% | 0.4% |  |
| skin | absent | 159 | 72 | 231 | 1.000 |
|  |  | 99.4% | 100.0% | 99.6% |  |
|  | present | 1 | 0 | 1 |  |
|  |  | 0.6% | 0.0% | 0.4% |  |
| total |  | 160 | 72 | 232 |  |
|  |  | 69.0% | 31.0% | 100.0% |  |

**Supplementary Table S3. Positive rates of crucial biomarkers in GC**

|  | **Variables** | **No. (%)** |
| --- | --- | --- |
| **Total** |  | **1,000 (100%)** |
| CLDN18.2 |  |  |
|  | Negative | 344 (34.4%) |
|  | Positive | 656 (65.6%) |
| PD-L1 |  |  |
|  | Negative (CPS<5) | 633 (63.3%) |
|  | Positive (CPS≥5) | 367 (36.7%) |
| PD-L1 |  |  |
|  | CPS<10 | 740 (74.0%) |
|  | CPS≥10 | 260 (26.0%) |
| PD-L1 |  |  |
|  | CPS<1 | 322 (32.2%) |
|  | CPS≥1 | 678 (67.8%) |
| HER2 |  |  |
|  | Negative | 933 (93.3%) |
|  | Positive | 67 (6.7%) |
| FGFR2 |  |  |
|  | Negative | 846 (84.6%) |
|  | Positive | 154 (15.4%) |
| EBV |  |  |
|  | Negative | 936 (93.6%) |
|  | Positive | 64 (6.4%) |
| MSI |  |  |
|  | MSS/MSI-L | 901 (90.1%) |
|  | MSI-H | 99 (9.9%) |

MSS, microsatellite stable; MSI-L, microsatellite instability-low; MSI-H, microsatellite instability-high
