# Supplementary material for: Clinicopathologic and molecular characterization of stages II-IV gastric cancer with Claudin 18.2 expression
Source: Oncologist. 2024 Sep 21;30(2):oyae238. doi: 10.1093/oncolo/oyae238 (PMC11881060; doi:10.1093/oncolo/oyae238)
Supplement: oyae238_suppl_Supplementary_Material [file oyae238_suppl_supplementary_material.zip › Supplementary Figure Legends.docx]

**Supplementary Figure Legends**

Supplementary Figure S1. Kaplan-Meier survival curves according to CLDN18.2 expression status in each treatment.

Supplementary Figure S2. Kaplan-Meier survival curves according to CLDN18.2 expression status in each stage.
