# Supplementary figures and images for: Clinicopathologic and molecular characterization of stages II-IV gastric cancer with Claudin 18.2 expression
Source: Oncologist. 2024 Sep 21;30(2):oyae238. doi: 10.1093/oncolo/oyae238 (PMC11881060; doi:10.1093/oncolo/oyae238)

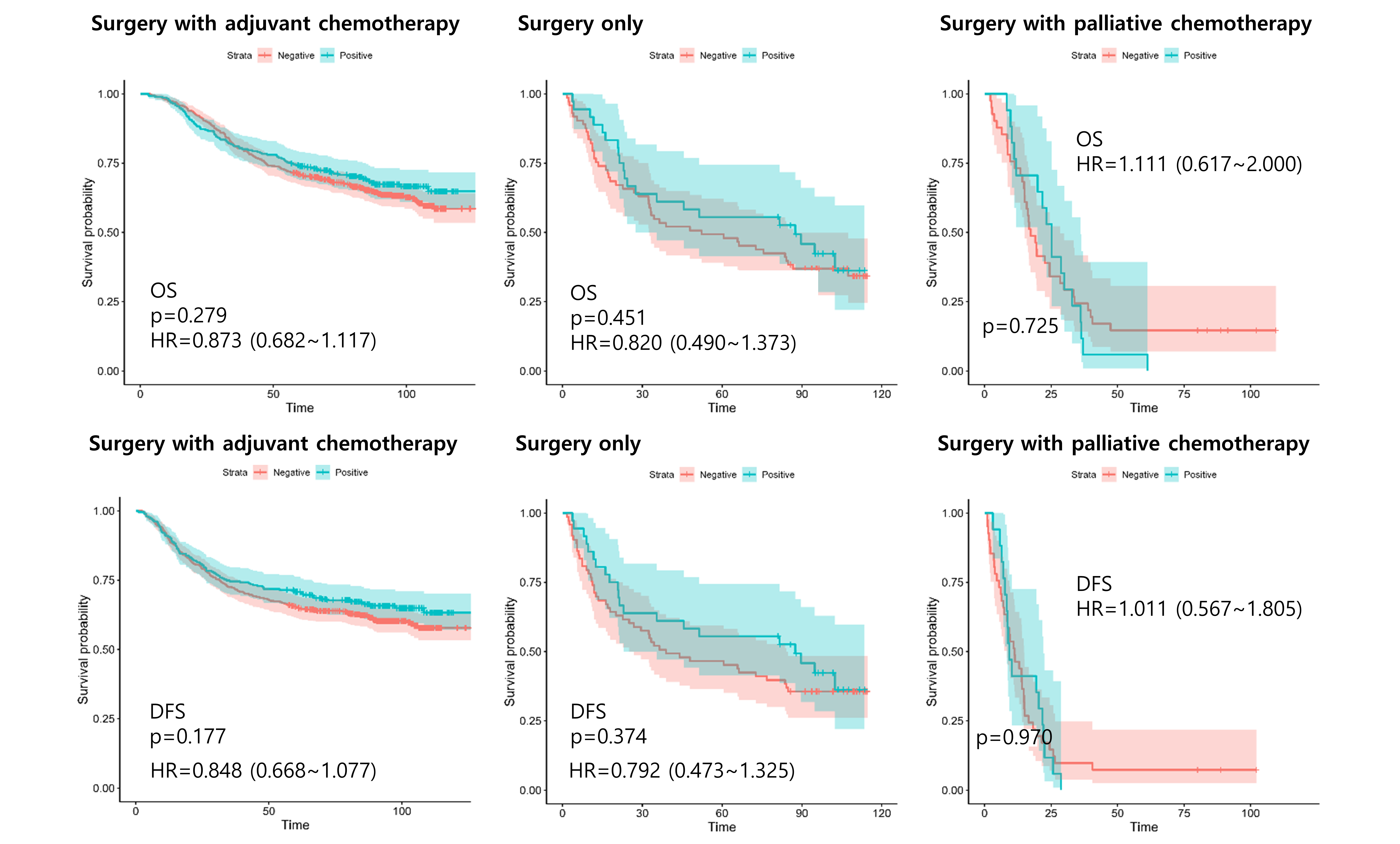

Supplement: oyae238_suppl_Supplementary_Material [file oyae238_suppl_supplementary_material.zip › Supplementary figure 1.TIF]

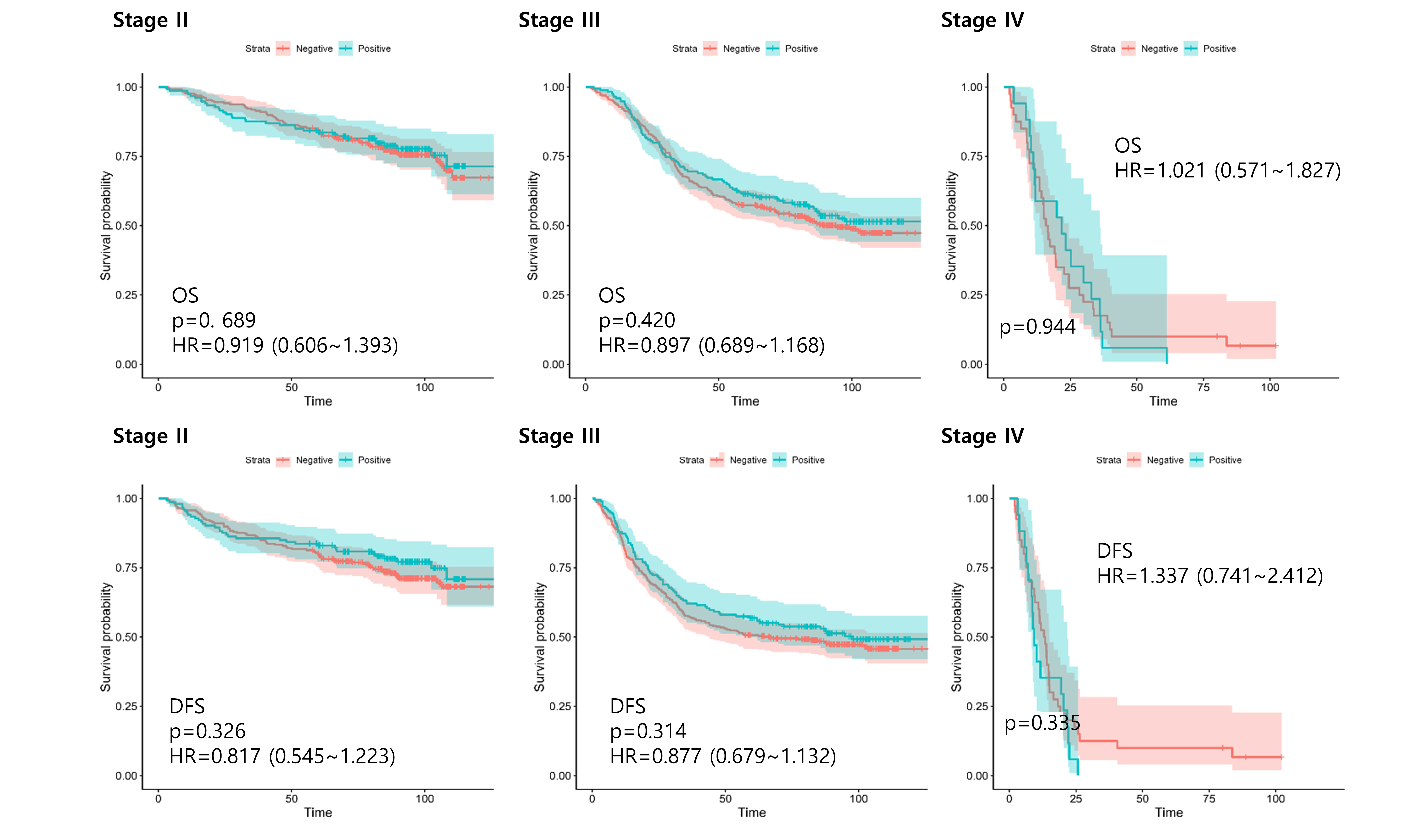

Supplement: oyae238_suppl_Supplementary_Material [file oyae238_suppl_supplementary_material.zip › Supplementary figure 2.TIF]
